# Association between Pretreatment Serum Uric Acid Levels and Progression of Newly Diagnosed Primary Angle-Closure Glaucoma: A Prospective Cohort Study

**DOI:** 10.1155/2019/7919836

**Published:** 2019-02-07

**Authors:** Shengjie Li, Mingxi Shao, Wenjun Cao, Xinghuai Sun

**Affiliations:** ^1^Department of Clinical Laboratory, Eye & ENT Hospital, Shanghai Medical College, Fudan University, Shanghai 200031, China; ^2^Department of Ophthalmology & Visual Science, Eye & ENT Hospital, Shanghai Medical College, Fudan University, Shanghai 200031, China; ^3^NHC Key Laboratory of Myopia (Fudan University), Key Laboratory of Myopia, Chinese Academy of Medical Sciences, Shanghai 200031, China; ^4^Shanghai Key Laboratory of Visual Impairment and Restoration (Fudan University), Shanghai 200031, China; ^5^State Key Laboratory of Medical Neurobiology, Institutes of Brain Science and Collaborative Innovation Center for Brain Science, Fudan University, Shanghai 200032, China

## Abstract

**Purpose:**

Increased evidence reveals that uric acid (UA) may have an important neuroprotective effect through its antioxidant properties. The aim of the present study was to investigate the relationship between pretreatment serum UA levels and the progression of newly diagnosed primary angle-closure glaucoma (PACG).

**Methods:**

This prospective observational cohort study included 64 patients with newly diagnosed PACG who were followed up for a mean period of 12.77 months (range: 3–28 months). All subjects underwent a complete ophthalmological examination during the baseline and final follow-up visits, together with the acquisition of blood samples for UA measurements. During the follow-up period, the progression of PACG was defined as a clinical diagnosis of medically uncontrolled intraocular pressure and a loss of visual field with a mean deviation of >1 dB/year. Univariable and multivariable Cox regression models were used to investigate the association between baseline serum UA levels and the progression of PACG. The cumulative probability of progression of glaucoma was analyzed using the Kaplan-Meier method.

**Results:**

During follow-up, 32 subjects were defined as progressive PACG, among whom baseline UA values were significantly higher in nonprogressing subjects than in progressing subjects (0.314 ± 0.069 mmol/l versus [vs.] 0.258 ± 0.069 mmol/l, respectively; *P* = 0.002). Similar results were also observed in male and female subgroups (*P* < 0.05). In a multivariable model, a decreased baseline serum UA level was associated with an increased risk for progressing PACG: both in male (hazard ratio [HR] 6.088 [95% confidence interval (CI) 1.163–31.8638]; *P* = 0.032) and female subjects (HR 3.565 [95% CI 1.131–11.236]; *P* = 0.030). Subjects with high UA levels demonstrated higher cumulative probabilities of nonprogressing PACG than those with low UA levels (male [16.67% vs. 80.00%; *P* = 0.0084] and female [29.41% vs. 68.00%; *P* = 0.0182]).

**Conclusion:**

An association between high baseline serum UA levels and a decreased risk for progressing PACG was found. This primary finding suggests that high serum UA levels may have a protective role against PACG and could slow disease progression.

## 1. Introduction

Uric acid (UA), the naturally occurring end-product of purine metabolism in humans, is a powerful water-soluble radical scavenger [1]. It does not only have the ability to block the generation of the strong oxidant peroxynitrite but also act as a chelator for metal ions (e.g., iron and copper) by converting them into their weak reactive forms, rendering them unable to catalyze free-radical reactions [1–3]. In addition, UA has been considered to be an advantageous evolutionary factor in early primates, which had increased levels of it [4, 5]. For example, uricase expression is 5- to >20-fold higher in humans than in most other mammals [6]. UA accounts for more than one-half of free-radical scavenging capacity in human blood, and there is increasing evidence demonstrating that UA plays a key role on account of its antioxidant properties [7, 8].

Recent studies have demonstrated potential roles for high levels of UA including reduced risk for cancer and related mortality [9, 10]; improved cognitive function [11, 12]; neuroprotective effects in stroke [13, 14]; and protection against neurodegenerative diseases such as multiple sclerosis [15, 16], Parkinson's disease [17, 18], and Alzheimer's disease [19]. In relation to our study, primary angle-closure glaucoma (PACG)—the most frequent cause of irreversible blindness worldwide [20]—is a chronic ocular neurodegenerative disease, characterized by visual field defects, optic nerve head cupping, and elevated intraocular pressure (IOP) [21]. Several previous investigations have reported that oxidative stress contributes to morphological and physiological alterations in the outflow of aqueous humor, subsequently causing damage to retinal ganglion cells in glaucoma [22–24]. Meanwhile, studies have reported that UA is one of the most important antioxidants in human biological fluids mitigating oxidative damage [8]. For example, a previous study reported significantly lower serum UA levels in patients with PACG [25] and primary open-angle glaucoma [26]. Additionally, these low levels were negatively associated with disease severity. In a study by Beit-Yannai et al., a significantly lower concentration of aqueous humor UA was observed in congenital glaucomatous rabbits [27]. Additionally, Knapp et al. [28] reported that serum UA levels were significantly lower in patients with optic neuritis than in normal (i.e., healthy) control subjects, further suggesting that UA may have a protective effect. Keller et al. [29] reported that UA could also protect hippocampal neurons against apoptosis by preventing mitochondrial superoxide accumulation and consequent peroxynitrite production and mitochondrial dysfunction. Overall, these studies suggest that higher serum UA levels could also have a similar protective role in PACG. However, it remains unclear whether UA is important in relation to the progression of PACG. Therefore, to obtain a potentially simple, rapid, and reliable prognostic parameter, this study investigated potential associations between pretreatment serum UA levels and the progression of PACG.

## 2. Materials and Methods

Participants in the present investigation were included in an observational cohort study (from January 1, 2016, to May 31, 2018) designed to evaluate the role of UA in PACG. The study was conducted at the Department of Ophthalmology and Visual Sciences, Eye and ENT Hospital of Fudan University, Shanghai, China. The Ethics Committee of the Eye and ENT Hospital approved this study, and fully informed consent was obtained from all subjects after explaining the conditions of this study. This study adhered to the principles of the Declaration of Helsinki.

Previously described diagnostic criteria for PACG [25, 30] were used to diagnose PACG. Briefly, PACG was diagnosed on the basis of narrow anterior chamber angles with glaucomatous optic neuropathy and corresponding visual field loss. This was determined using the following methods: a glaucoma hemifield test with outside normal limits that included a cluster of ≥3 nonedge contiguous points on the pattern deviation plot that crossed the horizontal meridian and had a probability of <5% of being present in age-matched healthy controls (one of which was <1%); an abnormal pattern standard deviation occurring with *P* < 5% in the normal population; and a completion of the test including the reliability criteria (fixation losses < 20%, false positives < 33%, and/or false negatives < 33%). Based on the results, PACG was diagnosed in eyes with narrow angles; an elevated IOP (>21 mmHg); at least 180 degrees of angle-closure eliminating the pigmented segment of the trabecular meshwork, regardless of whether synechial or appositional, segmented or continuous; and in eyes in which the degree of peripheral anterior synechiae was too extensive to be managed using laser peripheral iridotomy. The inclusion criteria for PACG subjects were as follows: ≥18 years of age; no systemic diseases such as acute infectious diseases, hyperuricemia, gout, metabolic syndrome, kidney disease, autoimmune disease, or cancer; no secondary glaucoma, any previous eye surgery, or any other eye disease that potentially could affect visual acuity or visual fields; newly diagnosed with PACG; and did not take medications that could influence serum UA levels.

During the baseline visit and final follow-up, the subjects underwent a complete ophthalmologic and medical examination [25, 26, 30]. This began with a standardized ophthalmologic examination performed by a glaucoma specialist. Gonioscopy was performed to determine the anterior chamber angle. IOP was measured three times using a Goldmann applanation tonometer (Haag-Streit, Bern, Switzerland), and the mean was calculated. Fundus photography was also performed using a digital retinal camera (TRC-NW200, Topcon, Tokyo, Japan); an A-scan ultrasound (A-Scan Pachymeter, Ultrasonic, Exton, PA, USA) was used to measure central corneal thickness (CCT), axial length (AL), and anterior chamber depth (ACD). The vertical cup-disk ratio (VCDR) was determined by analyzing the fundus photographs. Finally, the mean deviation (MD) and mean sensitivity (MS) of the visual fields were measured using automated Octopus perimetry (Haag-Streit, Switzerland). After considering the learning effect of the visual field tests, the results of the first two tests were excluded. Only reliable (i.e., false positive/negative < 15% and a reliability factor < 15) and compatible visual field results were included. Moreover, medical examinations—including assessments of electrocardiograms, X-rays, liver function, renal function, infectious disease, blood pressure, heart rate, body temperature, and body mass index (BMI)—were performed in all subjects at the Eye and ENT Hospital of Fudan University. Patients with diabetes mellitus and hypertension were defined by consistent self-reported history. Other information, such as age, sex, BMI, systolic blood pressure (SBP), diastolic blood pressure (DBP), liver function, renal function, heart rate, and body temperature, were also collected.

All patients underwent monthly follow-up by telephone communication to keep the investigators up-to-date on disease progress of PACG (the minimum follow-up period was set at 12 months). Subjects who required eye surgery, or those with any other ocular or systemic disease that could have affected the visual field, IOP, or UA levels, were excluded. During follow-up, subjects were classified into either progressing or nonprogressing groups.

**Figure 1:**
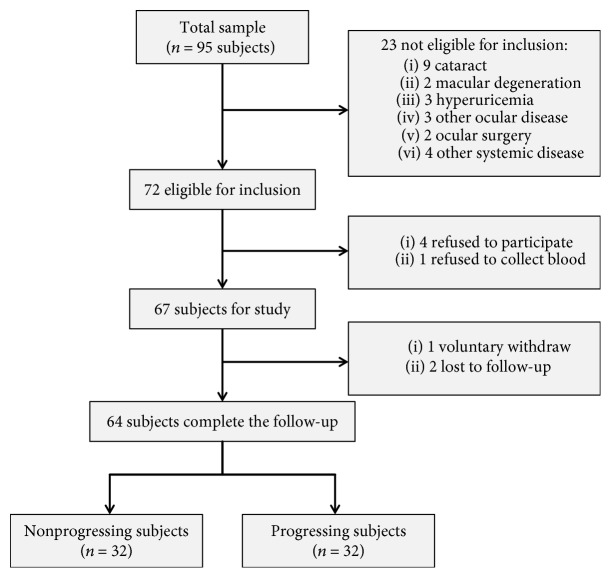
Study flow diagram.

Determination of functional PACG progression was based on two primary criteria [31, 32]. The first included the clinical diagnosis of medically uncontrollable IOP, which included all patients who received ≥1 topical glaucoma medication(s) during analysis, or in whom IOP was not lowered by at least 20% from the baseline IOP or was maintained at >21 mmHg. The second criterion was that the field of vision continued to decline (loss of visual field, with a mean deviation of >1 dB/year). However, subjects who were defined as progressive PACG *during* the follow-up period were also defined as such. Overall, 95 subjects who visited the Department of Ophthalmology and Visual Sciences between January 1, 2016, and May 31, 2018, were enrolled as PACG subjects in this study, of which 31 were later excluded (see the study cohort flow diagram in Figure 1).

During the baseline and final follow-up visits, various parameters were analyzed. For biochemical measurements, 4 ml of blood was obtained via standard venipuncture at the antecubital fossae (i.e., anterior elbow veins) on the morning after the subjects had fasted for 8 h and had refrained specifically from purine-rich foods (e.g., offal, seafood, and beans) for 2 days. The methods used to calculate UA levels have previously been described in detail [25, 26]. These studies also reported that UA level reference ranges vary in males (0.2023–0.4165 mmol/l) and females (0.1428–0.3392 mmol/l). Based on this, participants were categorized into subgroups according to sex in this study. Internal controls were analyzed daily over a 3-year period, with typical monthly coefficients of variation of 2–4% and no significant changes in UA levels.

All analyses were performed using SPSS version 13.0 (SPSS Inc., Chicago, IL, USA), and all figures were created using GraphPad Prism version 6 (GraphPad, La Jolla, CA, USA). Data are presented as mean ± standard deviation (SD). Normality was assessed using the Kolmogorov-Smirnoff test. Independent Student's *t*-test and chi-squared test were used to compare the characteristics of subjects between groups. A paired *t*-test was used for the comparison factors of the baseline and the follow-up measurements. Univariate and multivariate Cox regression analyses were used to analyze the association between baseline serum UA levels and disease progression of PACG. Furthermore, Cox proportional hazards models were used to obtain hazard ratios (HRs) and to identify baseline factors that predicted which subjects would be classified into the nonprogressing PACG group during the follow-up period. The cumulative incidence of nonprogressing PACG according to the serum UA level was assessed using Kaplan-Meier plots, and the log-rank test was used to assess differences between the curves. Results with two-sided *P* < 0.05 were considered to be statistically significant.

## 3. Results

A total of 64 PACG patients (22 male and 42 female) with a mean (±SD) age of 58.30 ± 15.24 years and a mean follow-up period of 12.77 months (range: 3–28 months) were included in the analysis. All patients received antiglaucoma medicine during the follow-up period. The demographic and clinical characteristics of the subjects are summarized in Table 1. Of the 64 PACG patients, 32 (12 male and 20 female) exhibited progressing PACG, and 32 (10 male and 22 female) exhibited nonprogressing PACG during the follow-up period. There was no statistical difference in age, sex, BMI, SBP, hypertension, diabetes, IOP, VCDR, CCT, ACD, MD, or MS between the progressing and nonprogressing patients (*P* > 0.05 for both) at baseline. However, the mean value of DBP and AL demonstrated significant differences at baseline between progressing and nonprogressing patients (*P* < 0.05).

**Figure 2:**
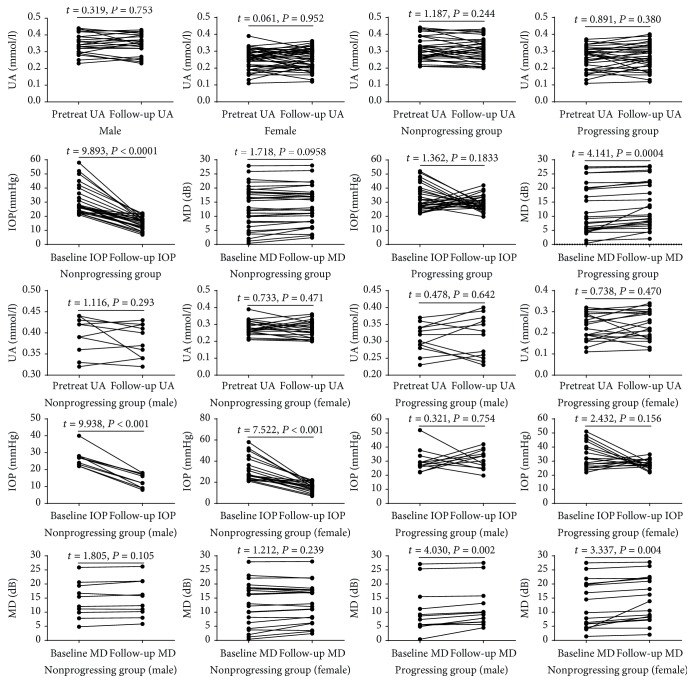
Comparison of serum uric acid (UA), intraocular pressure (IOP), and mean deviation (MD) levels at baseline and last follow-up visits. All patients underwent a monthly follow-up (the minimum follow-up period was set at 12 months). Each data point represents one subject. A paired *t*-test was used to compare factors at the baseline and the follow-up measurements.

The mean baseline UA levels in male and female patients were 0.347 ± 0.061 mmol/l and 0.254 ± 0.060 mmol/l, respectively. Additionally, the mean MD and IOP were 12.98 ± 7.93 dB and 31.12 ± 9.13 mmHg, respectively. There was no significant difference between pretreatment and follow-up UA levels in both male and female subjects (*P* > 0.05) (Figure 2). Moreover, there was also no significant difference between the pretreatment and follow-up UA levels in the progressing and nonprogressing PACG groups (*P* > 0.05) (Figure 2). In the progressing group, there was no significant difference between the baseline and follow-up IOP levels (*P* > 0.05) (Figure 2); however, MD levels were significantly higher in the final follow-up compared with baseline levels (*P* = 0.0004) (Figure 2). In contrast, the nonprogressing group exhibited no significant difference between baseline and follow-up MD levels (*P* > 0.05) (Figure 2); however, IOP values were significantly higher at the final follow-up compared with baseline (*P* < 0.0001) (Figure 2). Similar results were also reported in the male and female subgroups (Figure 2).

Baseline UA values were significantly higher in patients who were classified with nonprogressing glaucoma versus those who were classified with progressing glaucoma (0.314 ± 0.069 mmol/l vs. 0.258 ± 0.069 mmol/l; *P* = 0.002). Similar results were also observed in the male (0.347 ± 0.061 mmol/l vs. 0.308 ± 0.043 mmol/l; *P* < 0.001) and female (0.254 ± 0.060 mmol/l vs. 0.228 ± 0.065 mmol/l; *P* = 0.007) subgroups (Table 1). During the follow-ups, the UA values were also significantly higher in the nonprogressing glaucoma than in the progressing glaucoma (0.286 ± 0.074 mmol/l vs. 0.266 ± 0.075 mmol/l, *P* = 0.033) group. Similar results were also observed in the male (0.345 ± 0.062 mmol/l vs. 0.314 ± 0.061 mmol/l; *P* = 0.008) and female (0.255 ± 0.061 mmol/l vs. 0.237 ± 0.069 mmol/l, *P* = 0.071) subgroups (Table 1).

The results of the univariable and multivariate models, which were used to investigate factors associated with the progression of PACG, are shown in Table 2. In the univariable model, a lower pretreatment level of UA (as a risk factor) predicted the progression of PACG over a 12-month follow-up period in both male (HR 5.657 [95% CI 1.224–26.148]; *P* = 0.027) and female (HR 2.680 [95% CI 1.093–6.571]; *P* = 0.031) subjects. After adjusting the multivariable model for age, hypertension, diabetes, IOP, VCDR, CCT, ACD, AL, and MD, lower pretreatment levels of UA remained a risk factor that predicted the progression of PACG in both male (HR 6.088 [95% CI 1.163–31.8638]; *P* = 0.032) and female (HR 3.565 [95% CI 1.131–11.236]; *P* = 0.030) subjects.

**Figure 3:**
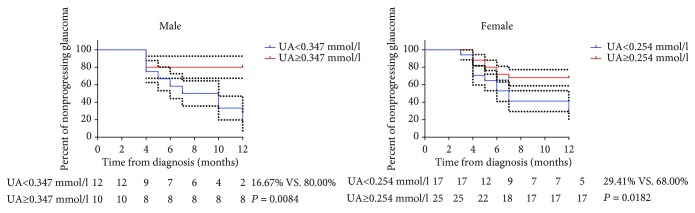
Kaplan-Meier curve stratified according to mean value of pretreatment uric acid (UA) levels (male: 0.347 mmol/l; female: 0.254 mmol/l) regarding survival for patients with primary angle-closure glaucoma. The log-rank test was used to calculate *P* values. The dashed line indicates the 95% confidence interval.

Figure 3 presents the cumulative probabilities of nonprogressing glaucoma with serum UA levels equal or greater than the average (male: 0.347 mmol/l; female: 0.254 mmol/l) and with serum UA levels less than average in male and female subjects (16.67% vs. 80.00%, *P* = 0.0084; 29.41% vs. 68.00%, *P* = 0.0182, respectively).

## 4. Discussion

In this prospective observational cohort study, we found that baseline serum UA levels were significantly higher in subjects with nonprogressing compared with progressing PACG. In contrast, patients with lower baseline serum UA levels had a higher probability of developing glaucomatous progression during the follow-up period. This association was also evident in the values obtained from the multivariable model (after adjustments for other factors). Similar results were also observed in the male and female subgroups, further supporting the protective effect of UA. Results of the current study are consistent with our previous case-control studies that demonstrated significantly decreased serum UA levels in glaucoma patients, which was negatively associated with disease severity [25, 26]. Collectively, our results reveal a potential protective effect of serum UA against the progression of PACG because lower levels of serum UA were associated with a higher risk for progression of PACG. To the best of our knowledge, the present investigation was the first prospective longitudinal study to assess the relationship between baseline serum UA levels and the progression of newly diagnosed PACG. Our findings suggest that high baseline serum UA levels may have a protective role in PACG and slow the progression of the disease. Moreover, these findings suggest that serum UA levels could be used as a novel marker for the progression of PACG.

To date, there has been increasing evidence suggesting that oxidative stress may be involved in the pathogenesis of glaucoma [33, 34]. UA is a well-known natural antioxidant present in several fluids and tissues of the human body [35], with Waring [36] reporting that UA may even contribute up to two-thirds of the antioxidant capacity of human blood. Fabbrini et al. [37] reported that subjects with a high UA level had 20–90% greater systemic nonenzymatic antioxidant capacity and lower levels (30%) of oxidative stress markers than those with lower UA levels. Furthermore, several studies have reported that the level of total antioxidant capacity in the aqueous humor and blood samples in patients with glaucoma was significantly reduced [38, 39]. For example, Tanito et al. [40] found that a lower systemic antioxidant capacity is associated with increased severity of visual field damage. Thus, these results suggest that lower serum UA levels are associated with increased severity of visual field damage.

In the literature, there are limited data regarding the association of serum UA levels and the progression of PACG. However, Aliena-Valero et al. [41] found that in a rat model of ischemic stroke, with rats subjected to UA treatment after a 7-day follow-up, the animals exhibited a reduction in neurofunctional impairment and infarct size compared with those treated with vehicle. Results of a prospective cohort study involving a large sample size (*n* = 12, 798) by Wang et al. [42] suggested that a higher baseline UA level was associated with better follow-up cognition among middle-age and older Chinese adults. Pellecchia et al. [43] reported that a lower baseline level of serum UA contributed to the occurrence of mild cognitive impairment after a 4-year follow-up of patients with early Parkinson's disease. Human clinical trials and experiments using animal models have suggested that increased UA levels can protect neurons against degeneration. The neuroprotective effects afforded by UA have mostly been attributed to upregulation of the glial glutamate transporter EAAT-1, a decrease in reactive oxygen species-dependent damage, and the modulation of glutathione synthesis [44, 45]. Thus, based on the existing literature, we speculate that lower levels of serum UA may accelerate disease progression in patients affected by PACG.

In a congenital glaucomatic rabbit model, UA levels in the aqueous humor have been found to be significantly lower compared with age-matched controls (17.1 ± 3.2 and 189.1 ± 75.70 *μ*M/mg, respectively) [27]. Several studies have also reported that UA levels in patients with neuromyelitis optica were significantly lower compared with healthy control subjects [46, 47]. With the focus more on eyes and their nerves, these findings directly support our results in that subjects with a lower baseline serum UA level have a higher probability of experiencing glaucomatous progression during the follow-up period. Therefore, a better understanding of serum UA levels and its possible role in PACG may be clinically useful for management of the disease. UA is a naturally occurring product of purine metabolism and is typically used as a biomarker for kidney disease. In fact, several previous studies have reported on the relationship between renal function and glaucoma. A study from the Korea National Health and Nutrition Examination Survey 2010–2011 reported that low estimated glomerular filtration rate levels are independently associated with glaucoma [48]. Additionally, Yuki et al. [49] found that UA levels were significantly higher in normal-tension glaucoma patients compared with controls. However, our focus was on the function of the antioxidant properties of UA and not on renal function. For this reason, subjects with hyperuricemia and gout were excluded, and there was no significant difference between baseline UA levels and follow-up UA levels, suggesting that antiglaucoma medicine did not affect kidney function.

Our previous study demonstrated that serum UA concentrations were 0.342 mmol/l (male) and 0.268 mmol/l (female) in normal control subjects [25]. In the present study, the mean serum UA level was 0.394 mmol/l (male) and 0.278 mmol/l (female) in the nonprogressing group and 0.308 mmol/l (male) and 0.228 mmol/l (female) in the progressing group. Moreover, the mean serum levels of UA were higher in the nonprogressing group than in normal control subjects. However, the mean serum levels of UA were lower in the progressing group than in normal control subjects. The increased serum levels of UA in the nonprogressing PACG patients would be consumed in the disease process by preferentially reacting with oxidizing agents to prevent damage from oxidative stress. These results suggest that decreased UA levels may be associated with an increased risk for PACG. In other words, high serum UA levels may have a protective role against the progression of PACG.

Although the present study was the first to investigate the relationship between pretreatment serum UA levels and the progression of newly diagnosed PACG, we acknowledge that the study had some limitations. First, this was a single-center study involving a relatively small sample size. Further studies with larger sample sizes and multicenter settings are required to further confirm our findings. Second, antiglaucoma therapy has been shown to prevent or delay the development of retinal ganglion cell damage. Although all subjects received antiglaucoma therapy in that study, our study did not collect detailed information on medicine usage for data analysis. Third, the average 12.77-month follow-up period may be considered to be relatively short for the evaluation of long-term effects of serum UA levels on glaucoma progression.

## 5. Conclusion

This prospective observational cohort study demonstrated that higher baseline serum UA levels were correlated with a decreased risk for the progression of newly diagnosed PACG. Together, these findings may support the concept that high serum UA levels could have a protective role in PACG and slow the progression of disease.

## Figures and Tables

**Table 1:** Demographic, ophthalmic characteristics, and serum UA levels of PACG.

Factors	PACG (*n* = 64)	Progressing group (*n* = 32)	Nonprogressing group (*n* = 32)	*P* value^∗^
Age, mean (SD) (years)	58.30 (15.24)	59.81 (15.19)	58.09 (15.27)	0.657
Sex, male/female	22/42	12/20	10/22	0.599
BMI, mean (SD) (kg/m^2^)	23.96 (3.24)	23.83 (3.16)	24.19 (3.46)	0.720
SBP, mean (SD) (mmHg)	129.29 (15.13)	127.94 (14.68)	131.61 (16.04)	0.418
DBP, mean (SD) (mmHg)	75.73 (10.78)	78.42 (9.42)	71.11 (11.66)	0.021
Diabetes, yes/no	8/56	5/27	3/29	0.708
Hypertension, yes/no	10/54	6/26	4/28	0.491
Baseline IOP, mean (SD) (mmHg)	31.12 (9.13)	32.17 (8.59)	30.10 (9.64)	0.372
Baseline VCDR, mean (SD)	0.60 (0.22)	0.59 (0.21)	0.64 (0.26)	0.449
Baseline CCT, mean (SD) (*μ*m)	550.47 (42.33)	554.10 (43.72)	542.08 (39.30)	0.399
Baseline ACD, mean (SD) (mm)	2.20 (0.76)	2.19 (0.78)	2.24 (0.74)	0.851
Baseline AL, mean (SD) (mm)	22.87 (1.43)	23.22 (1.51)	22.18 (0.98)	0.025
Baseline MD, mean (SD) (dB)	12.98 (7.93)	12.28 (8.73)	13.52 (7.35)	0.564
Baseline MS, mean (SD) (dB)	14.73 (8.18)	15.30 (9.16)	14.29 (7.43)	0.650
Pretreat UA, mean (SD) (mmol/l)	0.286 (0.075)	0.258 (0.069)	0.314 (0.069)	0.002
Pretreat UA (male), mean (SD) (mmol/l)	0.347 (0.061)	0.308 (0.043)	0.394 (0.044)	<0.001
Pretreat UA (female), mean (SD) (mmol/l)	0.254 (0.060)	0.228 (0.065)	0.278 (0.043)	0.007
Follow-up UA, mean (SD) (mmol/l)	0.286 (0.074)	0.266 (0.075)	0.305 (0.069)	0.033
Follow-up UA (male), mean (SD) (mmol/l)	0.345 (0.062)	0.314 (0.061)	0.381 (0.040)	0.008
Follow-up UA (female), mean (SD) (mmol/l)	0.255 (0.061)	0.237 (0.069)	0.271 (0.049)	0.071
Follow-up IOP, mean (SD) (mmHg)	21.99 (8.63)	29.30 (5.05)	14.90 (4.35)	<0.001
Follow-up MD, mean (SD) (dB)	13.94 (7.32)	14.00 (8.20)	13.89 (6.69)	0.956
Duration of follow-up, (range) (month)	12.77 (3~28)	6.16 (3~12)	20.91 (12~28)	<0.001
Treatment (% yes)	100	100	100	1.00

BMI: body mass index; SBP: systolic blood pressure; DBP: diastolic blood pressure; IOP: intraocular pressure; VCDR: vertical cup-disc ratio; CCT: central corneal thickness; ACD: anterior chamber depth; AL: axial length; MD: visual field mean deviation; MS: visual field mean sensitivity; SD: standard deviation; UA: uric acid. ^∗^Independent Student's *t*-test, Fisher exact test, and *x*^2^ tests were used for comparison of patient characteristics between the progressing group and nonprogressing group.

**Table 2:** Univariate and multivariate Cox proportional analysis for progression in patients with PACG.

	Univariate analysis		Multivariate analysis	
	HR (95% CI)	*P*	HR (95% CI)	*P*
*Male*				
UA ≥ 0.347	1		1	
UA < 0.347	5.657 (1.224-26.148)	0.027	6.088 (1.163-31.863)	0.032
*Female*				
UA ≥ 0.254	1		1	
UA < 0.254	2.680 (1.093-6.571)	0.031	3.565 (1.131-11.236)	0.030
*Gender*				
Male	1			
Female	1.135 (0.555- 2.322)	0.729	—	—
*Age*				
Age < 58.30	1			
Age ≥ 58.30	1.011 (0.495-2.064)	0.976	—	—
*Hypertension*				
No	1			
Yes	0.738 (0.303-1.795)	0.503	—	—
*Diabetes*				
No	1		1	
Yes	2.082 (0.899-4.826)	0.087	1.225 (0.405-3.711)	0.719
*IOP*				
IOP < 31.12	1		1	
IOP ≥ 31.12	1.682 (0.822-3.422)	0.155	1.573 (0.585-4.226)	0.369
*VCDR*				
VCDR < 0.6	1		1	
VCDR ≥ 0.6	1.690 (0.810-3.526)	0.162	1.275 (0.517-3.145)	0.598
*CCT*				
CCT< 550.47	1		1	
CCT ≥ 550.47	1.511 (0.732-3.122)	0.264	1.766 (0.532-5.862)	0.353
*ACD*				
ACD < 2.20	1			
ACD ≥ 2.20	1.051 (0.495-2.232)	0.897	—	—
*AL*				
AL < 22.87	1		1	
AL ≥ 22.87	1.579 (0.751-3.322)	0.229	1.691 (0.623-4.588)	0.303
*MD*				
MD < 12.98	1		1	
MD ≥ 12.98	1.439 (0.646-3.207)	0.373	1.334 (0.478-3.721)	0.582

IOP: intraocular pressure; VCDR: vertical cup-disc ratio; CCT: central corneal thickness; ACD: anterior chamber depth; AL: axial length; MD: visual field mean deviation; UA: uric acid.

## Data Availability

The data used to support the findings of this study are available from the corresponding author upon request.
